# Supplementary material for: Rapid wavefield forecasting for earthquake early warning via deep sequence to sequence learning
Source: Nat Commun. 2025 Nov 23;16:10622. doi: 10.1038/s41467-025-65435-2 (PMC12660841; doi:10.1038/s41467-025-65435-2)
Supplement: Supplementary file 1 — Supplementary Information [file 41467_2025_65435_MOESM1_ESM.pdf]

A Notation

Terms	Definition
WaveCastNet	Wavefield forecasting network (WaveCastNet) based on a seq2seq model.
Seq2Seq	AI-enabled sequence to sequence (seq2seq) modelling framework.
ConvLEM	Convolutional long expressive memory (ConvLEM) recurrent unit.
time window	Sequence length in temporal dimension.
arrival time	Time step at which maximal waveform arrives.
particle velocity	Single pixel $\mathbf{X}_t[c, h, w]$ in the snapshot.
waveform	Time series recorded for a single particle velocity.
wavefield	Snapshot $\mathbf{X}_t$ at a certain time step.
t	Temporal coordinate (time point).
h, w	XY-index of each input snapshot.
c	Channel index for velocity in a certain direction.
$\mathbf{X}_t$	Snapshot of shape $C \times H \times W$ at time step t.
$\mathbf{C}_n$	Fast hidden state in latent space.
$\mathbf{H}_n$	Slow hidden state in latent space.
X (NS)	North-South direction.
Y (EW)	East-West direction.
Z (UP)	Vertical direction, positive values signify upward movement.

Supplementary Table A.1: Terms and Definitions

B Technical Details

B.1 Discretized ConvLEM

Here we derive the discretized formula of ConvLEM from the following time-dependent ODEs:

$$\begin{aligned}\frac{d\mathbf{C}(t)}{dt} &= \psi_{\mathbf{C}}\left(\mathbf{C}(t), \mathbf{H}(t), \mathbf{X}(t)\right) = \mathbf{g}_c\left(\mathbf{H}(t), \mathbf{X}(t)\right) \odot \left[f^c_{\theta_c}(\mathbf{H}(t), \mathbf{X}(t)) - \mathbf{C}(t)\right], \\ \frac{d\mathbf{H}(t)}{dt} &= \psi_{\mathbf{H}}\left(\mathbf{C}(t), \mathbf{H}(t), \mathbf{X}(t)\right) = \mathbf{g}_h\left(\mathbf{H}(t), \mathbf{X}(t)\right) \odot \left[f^h_{\theta_h}(\mathbf{C}(t), \mathbf{X}(t)) - \mathbf{H}(t)\right].\end{aligned} \tag{1}$$

The gating functions $\mathbf{g}_c$, $\mathbf{g}_h$, and update functions $f^c_{\theta_c}$, $f^h_{\theta_h}$ are defined based on convolutional operation:

$$\begin{aligned}\mathbf{g}_c\left(\mathbf{H}, \mathbf{X}\right) &= \sigma\left(\mathbf{W}_{xt} * \mathbf{X} + \mathbf{W}_{ht} * \mathbf{H}\right), \\ \mathbf{g}_h\left(\mathbf{H}, \mathbf{X}\right) &= \sigma\left(\mathbf{W}_{x\bar{t}} * \mathbf{X} + \mathbf{W}_{h\bar{t}} * \mathbf{H}\right), \\ f^c_{\theta_c}(\mathbf{H}, \mathbf{X}) &= tanh\left(\mathbf{W}_{hc} * \mathbf{H} + \mathbf{W}_{xc} * \mathbf{X}\right), \\ f^h_{\theta_h}(\mathbf{C}, \mathbf{X}) &= tanh\left(\mathbf{W}_{ch} * \mathbf{C} + \mathbf{W}_{xh} * \mathbf{X}\right).\end{aligned} \tag{2}$$

In this notation, $\mathbf{H}(t)$ and $\mathbf{C}(t)$ denote the slow and fast evolving hidden states in latent space $\mathbb{R}^{r \times p \times q}$ respectively. $\mathbf{X}(t) \in \mathbb{R}^{c \times h \times w}$ represents a three-dimensional input tensor. $\mathbf{W}_{\cdot,\cdot}$ denotes the convolutional kernels, $\odot$ represents the Hadamard product, and $*$ indicates the convolutional operator. For brevity, bias vectors are omitted in gating and updated functions defined in 2.

We utilize the Implicit-Explicit (IMEX) time-stepping scheme to write the ODEs in Eq. (1) in a discretized formula, with subscript n as time steps index ranging from 1 to N. Given $\Delta t > 0$:

$$\begin{aligned}\frac{\mathbf{C}_n - \mathbf{C}_{n-1}}{\Delta t} &= \psi_{\mathbf{C}}\left(\mathbf{C}_{n-1}, \mathbf{H}_{n-1}, \mathbf{X}_n\right) = \mathbf{g}_c\left(\mathbf{H}_{n-1}, \mathbf{X}_n\right) \odot \left[f^c_{\theta_c}(\mathbf{H}_{n-1}, \mathbf{X}_n) - \mathbf{C}_{n-1}\right], \\ \frac{\mathbf{H}_n - \mathbf{H}_{n-1}}{\Delta t} &= \psi_{\mathbf{H}}\left(\mathbf{C}_n, \mathbf{H}_{n-1}, \mathbf{X}_n\right) = \mathbf{g}_h\left(\mathbf{H}_{n-1}, \mathbf{X}_n\right) \odot \left[f^h_{\theta_h}(\mathbf{C}_n, \mathbf{X}_n) - \mathbf{H}_{n-1}\right].\end{aligned} \tag{3}$$

For discretized fast hidden state $\mathbf{C}_n$, we have:

$$\begin{aligned}\mathbf{C}_n - \mathbf{C}_{n-1} &= \Delta t \cdot \mathbf{g}_c \odot (f^c_{\theta_c} - \mathbf{C}_{n-1}); \\ \mathbf{C}_n &= (\Delta t \cdot \mathbf{g}_c) \odot f^c_{\theta_c} + \mathbf{C}_{n-1} - (\Delta t \cdot \mathbf{g}_c) \odot \mathbf{C}_{n-1} \\ &= (\Delta t \cdot \mathbf{g}_c) \odot f^c_{\theta_c} + \mathbb{1} \odot \mathbf{C}_{n-1} - (\Delta t \cdot \mathbf{g}_c) \odot \mathbf{C}_{n-1} \\ &= (\Delta t \cdot \mathbf{g}_c) \odot f^c_{\theta_c} + (\mathbb{1} - \Delta t \cdot \mathbf{g}_c) \odot \mathbf{C}_{n-1},\end{aligned}$$

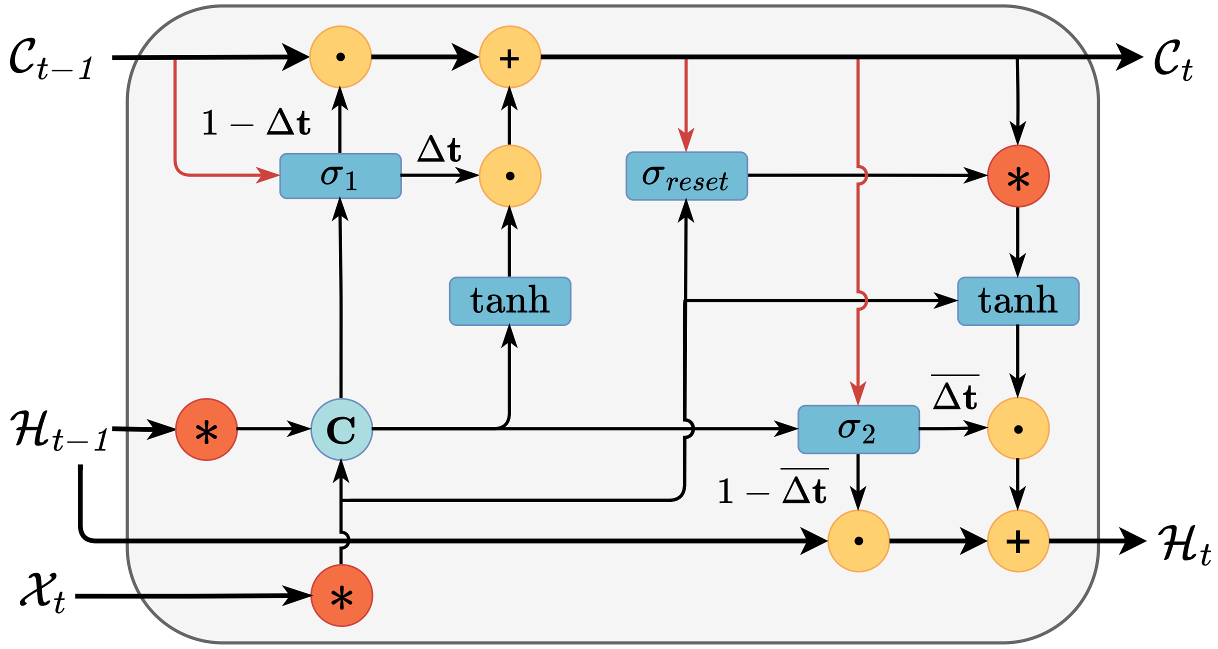

Supplementary Figure B.1: Schematic of the ConvLEM cell. Here, σ_1 and σ_2 represent $\mathbf{g}_c$ and $\mathbf{g}_h$ respectively. Update function f is set to tanh. Red links indicate peephole connections.

where $\mathbb{1}$ is the matrix of ones that matches the shape of hidden state $\mathbf{C}_n$ and $\mathbf{H}_n$.

Similarly, we have for $\mathbf{H}_n$:

$$\mathbf{H}_n = (\Delta t \cdot \mathbf{g}_h) \odot f^h_{\theta_h} + (\mathbb{1} - \Delta t \cdot \mathbf{g}_h) \odot \mathbf{H}_{n-1}.$$

Define $\mathbf{\Delta t}_n = \Delta t\, \mathbf{g}_c$, $\overline{\mathbf{\Delta t}_n} = \Delta t\, \mathbf{g}_h$. By plugging in 2 and 3, we derive the discretized formula for ConvLEM:

$$\begin{aligned}
\mathbf{\Delta t}_n &= \Delta t\, \mathbf{g}_c(\mathbf{H}_{n-1}, \mathbf{X}_n) \\
\overline{\mathbf{\Delta t}_n} &= \Delta t\, \mathbf{g}_h(\mathbf{H}_{n-1}, \mathbf{X}_n) \\
\mathbf{C}_n &= (\mathbb{1} - \mathbf{\Delta t}_n) \odot \mathbf{C}_{n-1} + \mathbf{\Delta t}_n \odot f^c_{\theta_c}(\mathbf{H}_{n-1}, \mathbf{X}_n) \\
\mathbf{H}_n &= \left(\mathbb{1} - \overline{\mathbf{\Delta t}_n}\right) \odot \mathbf{H}_{n-1} + \overline{\mathbf{\Delta t}_n} \odot f^h_{\theta_h}(\mathbf{C}_n, \mathbf{X}_n). \quad \square
\end{aligned} \tag{4}$$

Supplementary Figure B.1 illustrates the discretized ConvLEM unit.

B.2 Gating Function Distribution

We visualize the distribution of $\mathbf{\Delta t}$ and $\overline{\mathbf{\Delta t}}$ for the encoder ConvLEM cells in WaveCastNet on point-source small earthquakes in Supplementary Figure B.2. Here, we set the time step factor Δt in 4 to 1, so $\mathbf{\Delta t}$ and $\overline{\mathbf{\Delta t}}$ equal to the gating functions $\mathbf{g}_c$ and $\mathbf{g}_h$ for the hidden fast and slow states $\mathbf{C}(t)$ and $\mathbf{H}(t)$, respectively.

As shown in Supplementary Figure B.2, the observed occurrences of $\mathbf{\Delta t}$ and $\overline{\mathbf{\Delta t}}$ on each scale decay as a power law with respect to the amplitude of the scale [6].

By setting all axes to logarithmic scale, we can observe the different linear slopes and amplitude ranges for $\mathbf{\Delta t}$ and $\overline{\mathbf{\Delta t}}$. $\overline{\mathbf{\Delta t}}$ exhibits a smaller linear slope and a longer trailing tail, with a distribution at the amplitude closer to 0 compared to $\mathbf{\Delta t}$, allowing $\mathbf{H}(t)$ to better capture low-frequency features. In contrast, $\mathbf{\Delta t}$ is more centrally distributed near 1, showing a smaller amplitude range and a larger linear slope, reflecting the rapid change in the hidden state $\mathbf{C}(t)$. These observations prove that the temporal multiscale resolution structure of ConvLEM is essential for modeling the fast-slow dynamical pattern in ground-motion data.

B.3 Structure of Embedding and Reconstruction Layers

Here, we discuss the embedding and reconstruction layers.

The embedding layer for densely and regularly sampled inputs $x \in \mathbb{R}^{3\times344\times224}$ is composed of three cascaded encoder layers. A standard encoder layer comprises a convolutional layer, with kernel size $=(4,4)$, stride=2, padding=1, followed by a LeakyRelu activation layer and BatchNorm layer. Each encoder layer reduces the input spatial dimensions by a factor

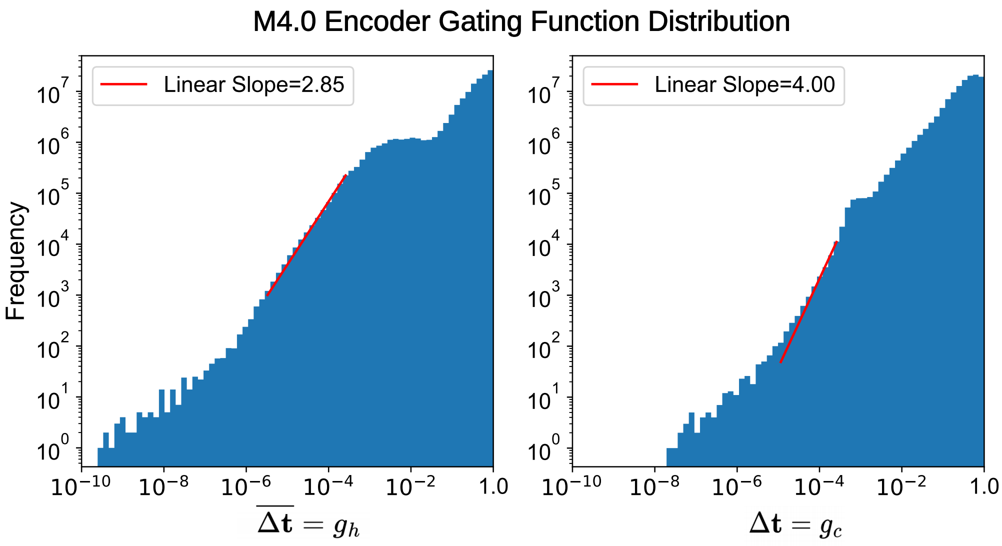

Supplementary Figure B.2: Histogram of $\mathbf{\Delta t}$ and $\overline{\mathbf{\Delta t}}$ for the encoder ConvLEM cells in WaveCastNet.

of 2. After the input signal passes through three encoder layers, the dimensions change from $3 \times 344 \times 224$ to a fixed-size latent space of $144 \times 43 \times 28$. The channel transformation process is illustrated in Supplementary Figure B.3.

The embedding layer for sparsely and irregularly sampled data maps the inputs $x \in \mathbb{R}^{3\times564}$ to a latent space of dimension $144 \times 43 \times 28$. Specifically, this embedding layer uses a shallow multilayer feedforward network [3], followed by two convolutional layers, as illustrated in Supplementary Figure B.3.

The reconstruction layer retrieves the predicted wavefield snapshot, shaped $144 \times 43 \times 28$, from the latent space. This process involves increasing spatial dimensions by a factor of 2 through transposed convolution, followed by a PixelShuffle layer [7] to further upscale the output by a factor of 4. The dimensional transformation process is depicted in Supplementary Figure B.3.

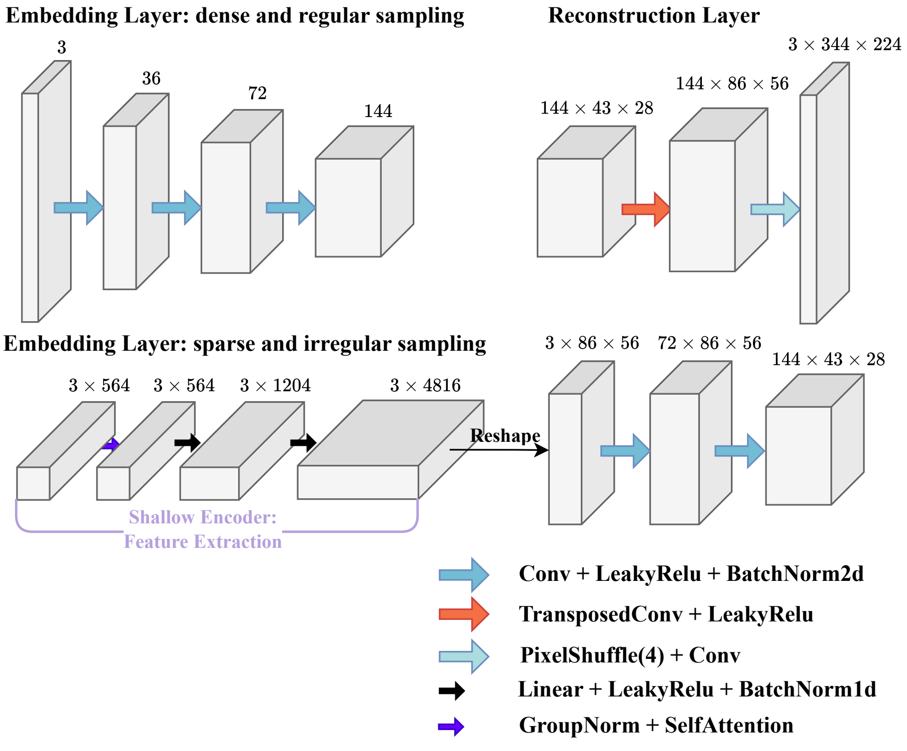

Supplementary Figure B.3: Detailed structure for the embedding layers and reconstruction layer in dense and sparse sampling scenarios.

B.4 Other Related Methods

We implemented ConvLSTM and ConvGRU with peephole connections as follows. For brevity, bias vectors are omitted from the activation and gating functions.

ConvLSTM

$$\begin{aligned}
\mathbf{i}_n &= \sigma\left(\mathbf{W}_{xi} * \mathbf{X}_n + \mathbf{W}_{hi} * \mathbf{H}_{n-1} + W_{ci} \odot \mathbf{C}_{n-1}\right), \\
\mathbf{f}_n &= \sigma\left(\mathbf{W}_{xf} * \mathbf{X}_n + \mathbf{W}_{hf} * \mathbf{H}_{n-1} + \mathbf{W}_{cf} \odot \mathbf{C}_{n-1}\right), \\
\mathbf{C}_n &= \mathbf{f}_n \odot \mathbf{C}_{n-1} + \mathbf{i}_n \odot f\left(\mathbf{W}_{xc} * \mathbf{X}_n + \mathbf{W}_{hc} * \mathbf{H}_{n-1}\right), \\
\mathbf{o}_n &= \sigma\left(\mathbf{W}_{xo} * \mathbf{X}_n + \mathbf{W}_{ho} * \mathbf{H}_{n-1} + \mathbf{W}_{co} \odot \mathbf{C}_n\right), \\
\mathbf{H}_n &= \mathbf{o}_n \odot f\left(\mathbf{C}_n\right).
\end{aligned}$$

ConvGRU

$$\begin{aligned}
\mathbf{Z}_n &= \sigma\left(\mathbf{W}_{xz} * \mathbf{X}_n + \mathbf{W}_{hz} * \mathbf{H}_{n-1}\right), \\
\mathbf{R}_n &= \sigma\left(\mathbf{W}_{xr} * \mathbf{X}_n + \mathbf{W}_{hr} * \mathbf{H}_{n-1}\right), \\
\mathbf{o}_n &= f\left(\mathbf{W}_{xo} * \mathbf{X}_n + \mathbf{R}_n \odot \left(\mathbf{W}_{ho} * \mathbf{H}_{n-1}\right)\right), \\
\mathbf{H}_n &= \left(1 - \mathbf{Z}_n\right) \odot \mathbf{H}_{n-1} + \mathbf{Z}_n \odot \mathbf{o}_n.
\end{aligned}$$

C Details on Training, Validation, and Test Sets

C.1 Post-Rupture Forecasting Accuracy

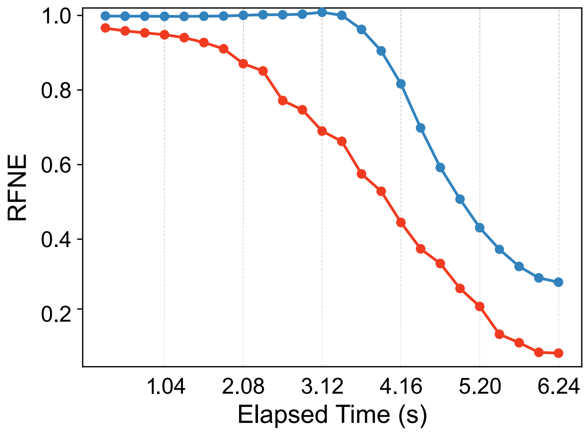

Supplementary Figure C.1: Forecasting accuracy as a function of time since rupture onset. The plot shows prediction errors in the waveforms (blue) and peak ground velocity (PGV) values (red) as the length of available post-rupture input increases. WaveCastNet remains effective even when provided with less than 15.6 seconds of initial input. Accurate forecasts are achieved with as little as 5.7 seconds of post-rupture data by padding shorter input sequences with white noise.

C.2 Configuration details

In Supplementary Table C.1, we summarize the details of the settings used for the tests. The split into training and testing data is determined based on epicenter locations: 80% of the events at each depth are randomly assigned to the training set, and the remaining 20% were assigned to the testing set. This selection process is conducted independently for each depth.

D Additional Experimental Results

D.1 Domain-shifted Settings

Noise tests To evaluate our WaveCastNet's robustness to noisy inputs, we augment our test data with two types of noise:

- **Empirical Noise:** This noise was designed to preserve the network-scale correlations observed in real-world data. The empirical noise had the shape $C \times T$(Input Length) $\times N$(Number of Stations), where it was directly added to the original waveforms to simulate realistic noise patterns that occur in practice.
- **Gaussian Noise:** To further stress-test the model, we also applied Gaussian noise with shape $C \times T \times N$, where the noise values were sampled from a normal distribution $N(\mu = 0, \sigma = 0.32)$. The standard deviation σ of the

Experiment	Input Resolution	T_{elapsed} (s)	T_{pred} (s)
Dense Sampling	344×224	5.7	101.4
Sparse Sampling	101	5.7	101.4
Noise Test	101	5.7	101.4
Latency Test	101	5.7	101.4
Source Location Test	344×224	5.7	101.4
Large M Test	344×224	15.6	101.4
Real world Data	178	15.6	15.6

Supplementary Table C.1: Configurations for different test setups. The prediction resolution is 344×224. T_{elapsed} denotes the time interval between the earthquake onset and the start of the prediction, and T_{pred} indicates the duration of a single one-time prediction inference. Specifically, a rolling prediction strategy is applied in real world scenarios, where 15.6-second forward predictions are performed at successive time intervals.

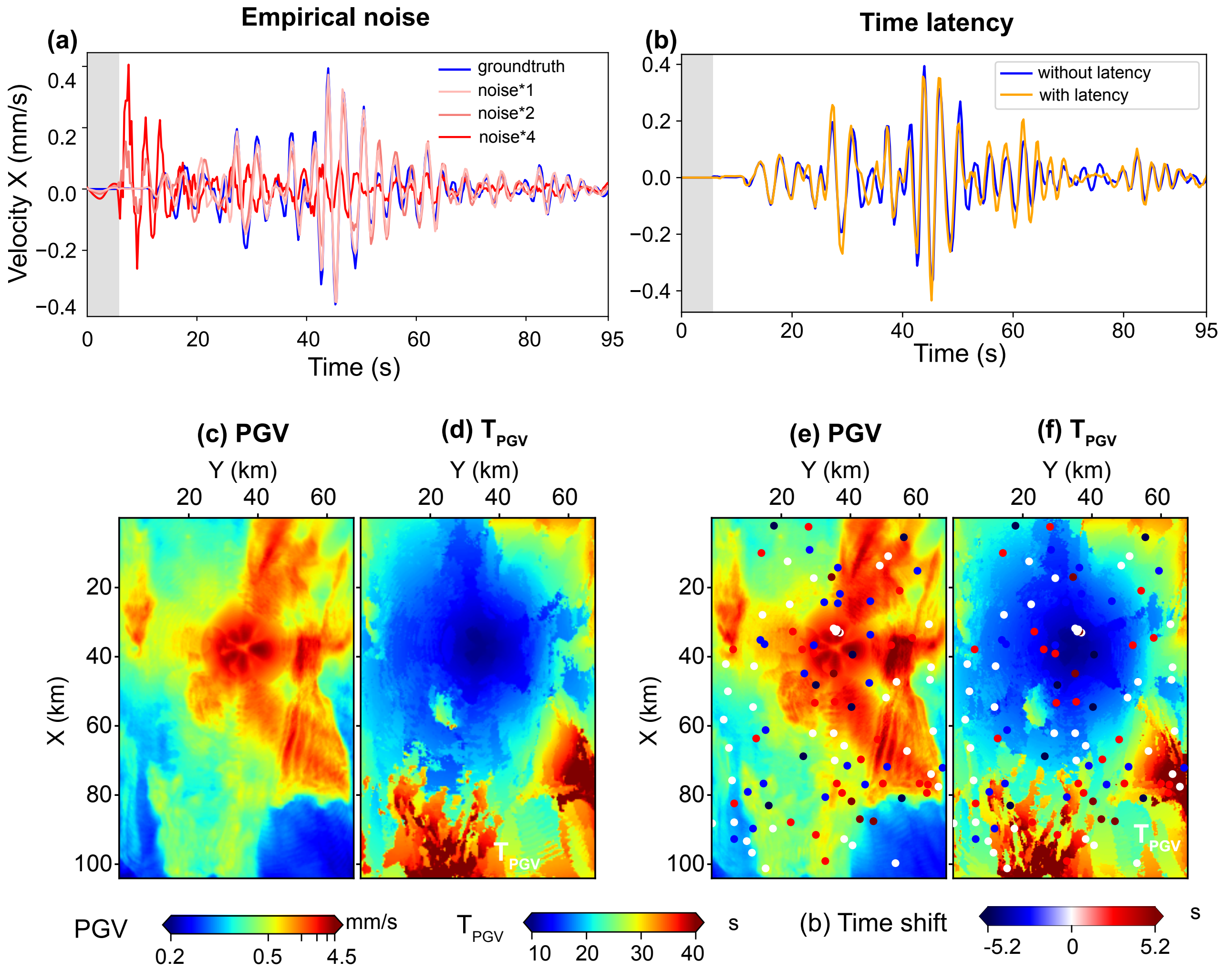

Supplementary Figure D.1: Noise (a,c,d) and time latency (b,e,f) tests. (a,b) X component waveforms at the San Jose station and (c,e) PGV (left) and (d,f) T_{PGV} maps. The gray shades in (a,b) indicate the input time window, and the circles in (f) the time shifts introduced at each station, respectively.

Gaussian noise was chosen after several iterations to match the performance degradation observed with the empirical noise. This allowed us to evaluate the behavior of the model at varying levels ν of noise perturbation (that is, the noise level is a multiplicative factor $\sigma\nu$).

For both noise types, we use the ShakeAlert station lists, and use a sparse model trained without any noise injection to ensure the evaluations were realistic. Our results, shown in Supplementary Figure D.2, demonstrate that the model maintains over 80% accuracy for both noise types at noise levels up to a factor of 2. This indicates that the model is resilient and can still perform well under moderate noise perturbations.

As illustrated in Supplementary Figures D.1(a), introducing empirical (station-specific) noise causes minimal performance degradation. We then scaled this noise by factors of 2 and 4 to simulate increasingly noisy scenarios. Even under these high-noise conditions, WaveCastNet effectively denoises and predicts the waveforms while accurately predicting PGVs and T_{PGV}. However, its performance starts to degrade when the noise multiplier exceeds 4. This desirable robustness can be attributed to the Seq2Seq architecture, which encodes inputs into a compressed latent representation before decoding them. The compressed latent state creates an information bottleneck: Only the most relevant information is retained in the latent state, while irrelevant details, such as noise or spurious signals, are discarded.

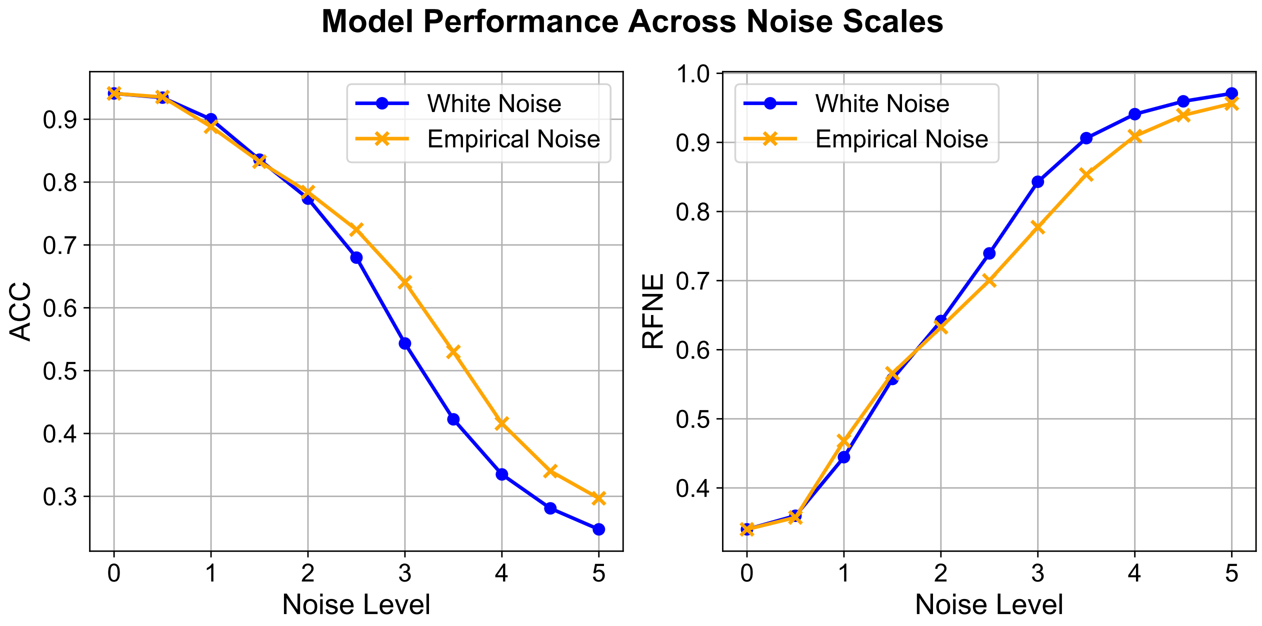

Supplementary Figure D.2: Evaluation metrics of WaveCastNet under varying levels of noise perturbation.

Uneven latency across the station. Real data transmission often results in uneven latencies across different stations. Asynchronous data ingestion can be addressed by using a buffer or interpolation mechanism to align incoming measurements with minimal delay. To simulate such conditions, we introduce random time shifts in sparse sampling scenarios (see Supplementary Figure D.1b and Section 4.5 in main text for details). The maximum time shift between stations is set to one second (equivalent to four discrete time steps), under which performance remains largely unaffected. However, when the maximum time shift exceeds two seconds, we observe a drop in accuracy.

D.2 Comparisons with an empirical ground motion model

To further validate the real-data results, we compare them against an ergodic ground motion model (GMM) using the ASK14 model from the NGA-West2 project [1]. Because our data are limited to a maximum frequency of 0.5,Hz, we evaluate spectral acceleration rather than PGVs. The PGVs calculated from this frequency range (<0.5 Hz) is lower than those predicted from the GMM that includes an entire frequency range. Supplementary Figure D.3 shows the spectral acceleration at 3 seconds of the GMM, our predicted values, and the observed waveforms. Since WaveCastNet does not generate predictions for the first 15.6 seconds, we calculate the spectral acceleration of the remaining portion of the signal. As a result, early arrival waveforms are not captured for near- to intermediate-distance stations (less than 30 km), causing underestimation relative to both the GMM and observed data. However, at larger distances, our predictions converge more closely with the observation, illustrating WaveCastNet's ability to capture non-ergodic variations in ground-motion intensities.

D.3 Comparative Study

Supplementary Table D.1 provides detailed configurations for the comparative studies.

D.4 Moving MNIST

Here, we show experiments for the MovingMNIST dataset to further demonstrate ConvLEM's performance in spatio-temporal forecasting. The MovingMNIST dataset [9] is a well-established benchmark for video prediction and spatiotemporal modeling tasks. This dataset consists of a total of $10,000$ videos, each comprising 20 fixed-size frames with dimensions of $1 \times 64 \times 64$ pixels. Each video sequence features two handwritten digits selected from the original MNIST dataset, which

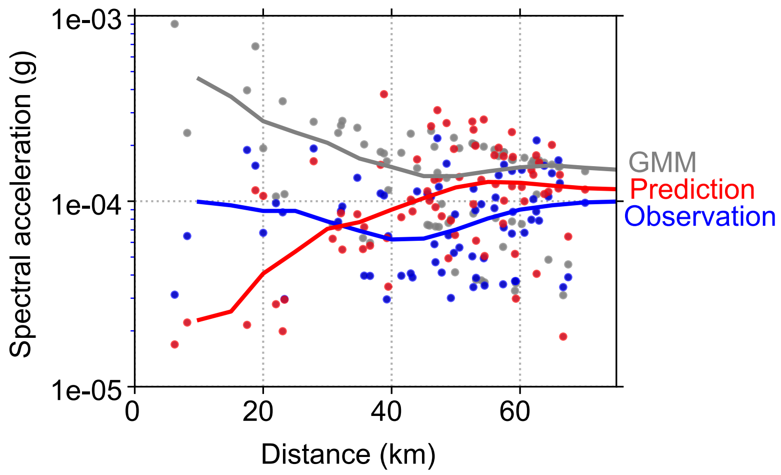

Supplementary Figure D.3: Comparisons of the spectral acceleration at 3 sec with respect to distances between (blue) real-world data, (red) WaveCastNet predictions and (gray) empirical ground motion model predictions. Circles indicate the calculated spectral accelerations, and solid lines indicate their median values computed for bins of 10 km

**Model**	**Parameters**	**Latent Space**	**Patch Size**	**Embed Dimension**	**ACC**	**RFNE**
Seq2Seq using ConvGRU[2]	4.99M	(144, 21, 14)	-	-	0.94	0.34
Seq2Seq using ConvLSTM [8]	6.65M	(144, 21, 14)	-	-	0.95	0.32
WaveCastNet (ours)	8.15M	(144, 21, 14)	-	-	0.96	0.27
Swin Transformer [5]	13.72M	-	(3,4,4)	144	0.95	0.31
Time-S-Former [4]	10.21M	-	(1,8,8)	192	0.95	0.31
Swin Transformer*	24.27M	-	(4,4,4)	192	0.97	0.25
Time-S-Former*	33.82M	-	(1,8,8)	192	0.98	0.20

Supplementary Table D.1: Performance comparison between Seq2Seq frameworks using different recurrent cells, and state-of-the-art transformers for forecasting small point-source earthquakes. While larger vision transformers can perform better on this task, we show that these models fail to generalize to domain-shifted settings in main-text Figure 9.

move within the frame at various speeds and directions. The digits exhibit diverse velocities and trajectories, including linear motion, bouncing off the frame edges, and occasional overlap, presenting a complex and challenging scenario for spatiotemporal forecasting models. The diversity in motion patterns makes the MovingMNIST dataset an ideal benchmark for evaluating the ability of models to capture and predict dynamic changes over time.

We use the first 10 frames as input to predict the subsequent 10 frames. The models consist of 3 stacked recurrent layers with no embedding or reconstruction layers involved. Supplementary Table D.2 shows the results for this task. Although using fewer parameters, ConvLEM is able to outperform the prediction performance of the ConvLSTM model. This further demonstrates ConvLEM's potential for spatio-temporal forecasting tasks.

**Model**	**Parameters**	**Latent Space**	**Layers**	**BCELoss** ↓
Stacked ConvLSTM	3.10M	(64, 64, 64)	3	206.13
Stacked ConvLEM	2.31M	(64, 64, 64)	3	166.75

Supplementary Table D.2: Results for Moving Mnist. The ConvLEM demonstrates improved forecasting capabilities while requiring fewer parameters than ConvLSTM.

E Kinematic rupture models

We present example kinematic rupture models for large earthquakes as in

- Supplementary Figure E.1: Kinematic rupture model of the M4.5 earthquake
- Supplementary Figure E.2: Kinematic rupture model of the M5 earthquake
- Supplementary Figure E.3: Kinematic rupture model of the M5.5 earthquake

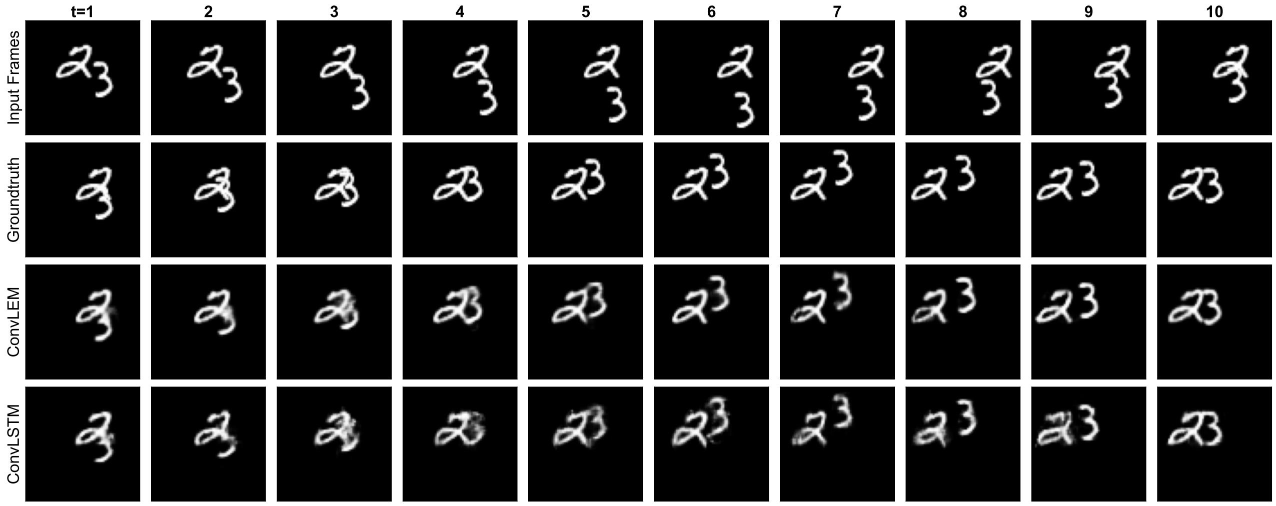

Supplementary Figure D.4: An example on MovingMnist dataset.

- Supplementary Figure E.4: Kinematic rupture model of the M6 earthquake
- Supplementary Figure E.5: Kinematic rupture model of the M6.5 earthquake
- Supplementary Figure E.6: Kinematic rupture model of the M7 earthquake

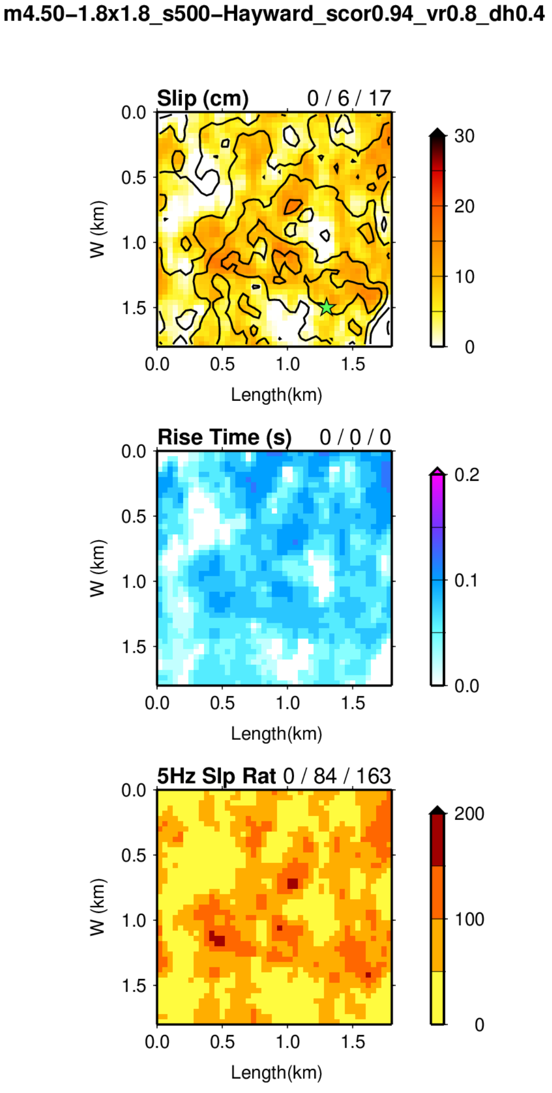

Supplementary Figure E.1: Kinematic rupture models for the M4.5 earthquake. (Top) slip (middle) rise time and (bottom) 5 Hz slip rate distributions.

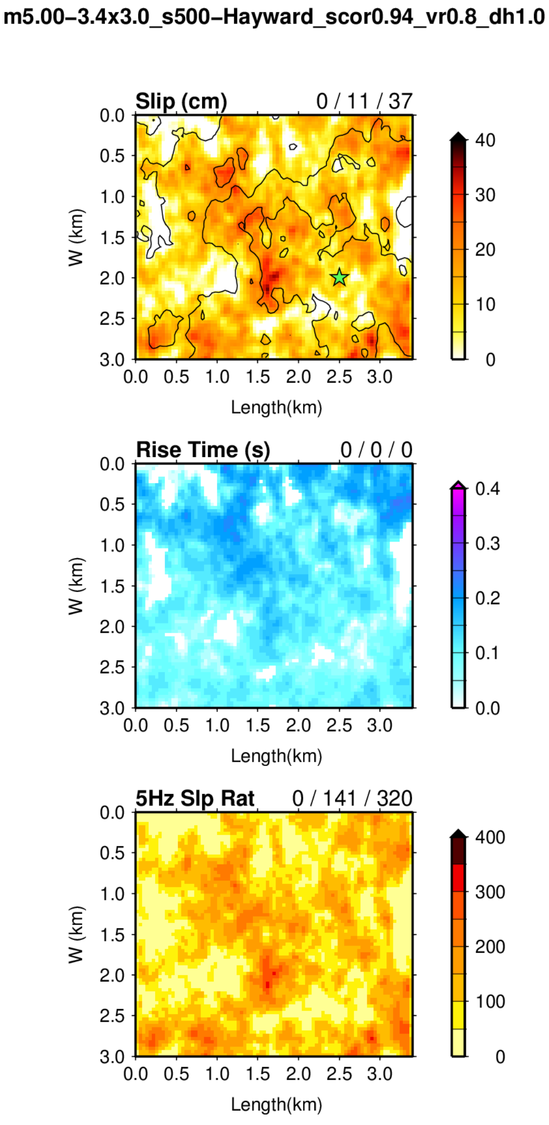

Supplementary Figure E.2: Kinematic rupture models for the M5.0 earthquake. (Top) slip (middle) rise time and (bottom) 5 Hz slip rate distributions.

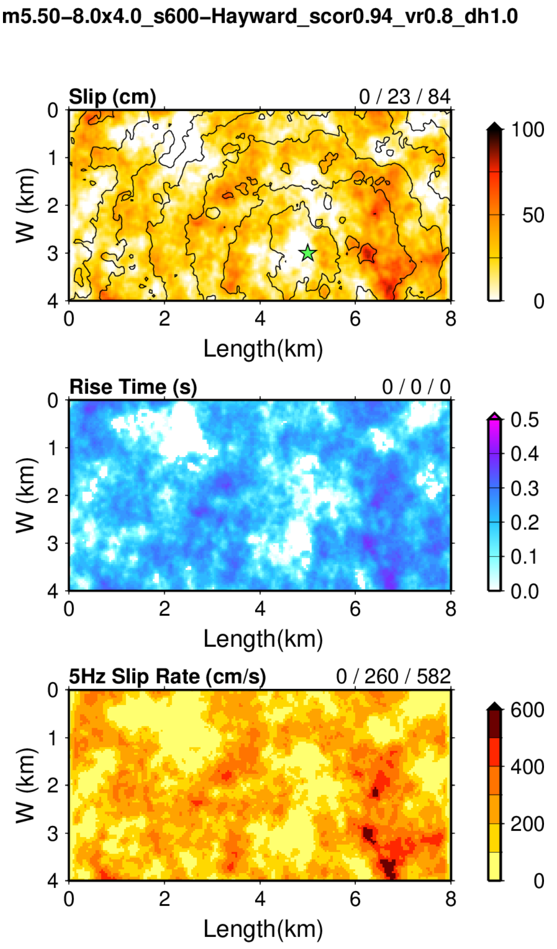

Supplementary Figure E.3: Kinematic rupture models for the M5.5 earthquake. (Top) slip (middle) rise time and (bottom) 5 Hz slip rate distributions.

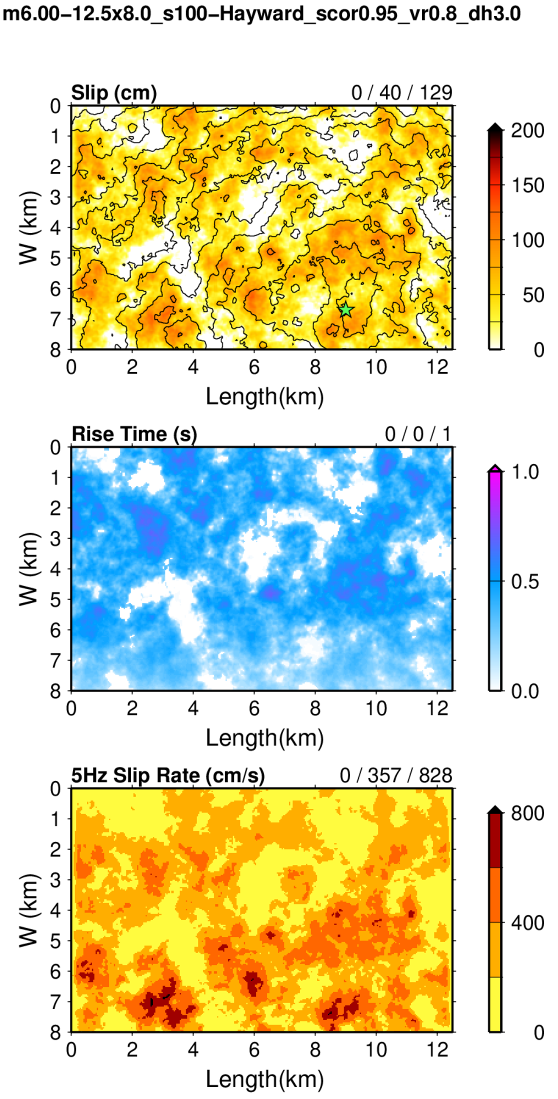

Supplementary Figure E.4: Kinematic rupture models for the M6 earthquake. (Top) slip (middle) rise time and (bottom) 5 Hz slip rate distributions.

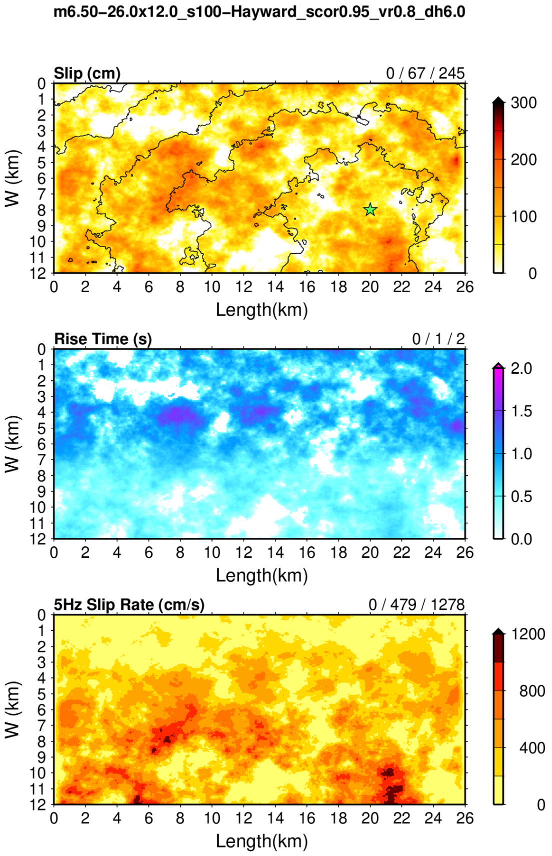

Supplementary Figure E.5: Kinematic rupture models for the M6.5 earthquake. (Top) slip (middle) rise time and (bottom) 5 Hz slip rate distributions.

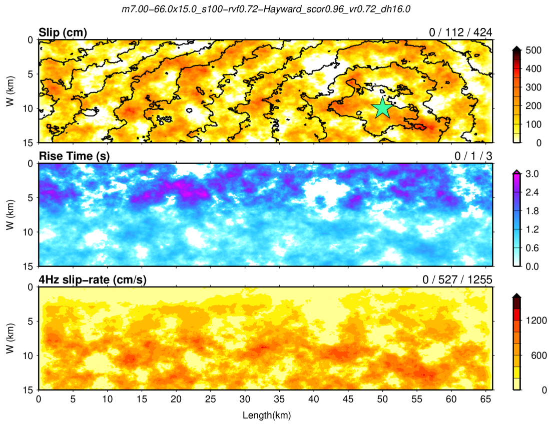

Supplementary Figure E.6: Kinematic rupture models for the M7 earthquake.(Top) slip (middle) rise time and (bottom) 5 Hz slip rate distributions.

References

[1] N. A. Abrahamson, W. J. Silva, and R. Kamai. Summary of the ASK14 Ground Motion Relation for Active Crustal Regions. *Earthquake Spectra*, 30(3):1025–1055, 2014.

[2] N. Ballas, L. Yao, C. Pal, and A. Courville. Delving Deeper Into Convolutional Networks for Learning Video Representations. *International Conference on Learning Representations*, 2016.

[3] N. B. Erichson, L. Mathelin, Z. Yao, S. L. Brunton, M. W. Mahoney, and J. N. Kutz. Shallow Neural Networks for Fluid Flow Reconstruction With Limited Sensors. *Proceedings of the Royal Society A*, 476(2238):20200097, 2020.

[4] Gedas Bertasius and Heng Wang and Lorenzo Torresani. Is Space-Time Attention All You Need for Video Understanding? In *International Conference on Machine Learning*, 2021.

[5] Z. Liu, J. Ning, Y. Cao, Y. Wei, Z. Zhang, S. Lin, and H. Hu. Video Swin Transformer. In *Proceedings of the IEEE/CVF Conference on Computer Vision and Pattern Recognition*, pages 3202–3211, 2022.

[6] T. K. Rusch, S. Mishra, N. B. Erichson, and M. W. Mahoney. Long Expressive Memory for Sequence Modeling. In *International Conference on Learning Representations*, 2021.

[7] W. Shi, J. Caballero, F. Huszár, J. Totz, A. P. Aitken, R. Bishop, D. Rueckert, and Z. Wang. Real-Time Single Image and Video Super-Resolution Using an Efficient Sub-Pixel Convolutional Neural Network. In *Proceedings of the IEEE/CVF Conference on Computer Vision and Pattern Recognition*, pages 1874–1883, 2016.

[8] X. Shi, Z. Chen, H. Wang, D.-Y. Yeung, W.-K. Wong, and W.-c. Woo. Convolutional LSTM Network: A Machine Learning Approach for Precipitation Nowcasting. *Conference on Neural Information Processing Systems*, 28, 2015.

[9] N. Srivastava, E. Mansimov, and R. Salakhudinov. Unsupervised Learning of Video Representations Using LSTMs. In *International Conference on Machine Learning*, pages 843–852, 2015.
